# Possible Involvement of TLRs and Hemichannels in Stress-Induced CNS Dysfunction via Mastocytes, and Glia Activation

**DOI:** 10.1155/2013/893521

**Published:** 2013-07-02

**Authors:** Adam Aguirre, Carola J. Maturana, Paloma A. Harcha, Juan C. Sáez

**Affiliations:** ^1^Departamento de Fisiología, Pontificia Universidad Católica de Chile, Santiago, Chile; ^2^Instituto Milenio, Centro Interdisciplinario de Neurociencias de Valparaíso, Valparaíso, Chile

## Abstract

In the central nervous system (CNS), mastocytes and glial cells (microglia, astrocytes and oligodendrocytes) function as sensors of neuroinflammatory conditions, responding to stress triggers or becoming sensitized to subsequent proinflammatory challenges. The corticotropin-releasing hormone and glucocorticoids are critical players in stress-induced mastocyte degranulation and potentiation of glial inflammatory responses, respectively. Mastocytes and glial cells express different toll-like receptor (TLR) family members, and their activation via proinflammatory molecules can increase the expression of connexin hemichannels and pannexin channels in glial cells. These membrane pores are oligohexamers of the corresponding protein subunits located in the cell surface. They allow ATP release and Ca^2+^ influx, which are two important elements of inflammation. Consequently, activated microglia and astrocytes release ATP and glutamate, affecting myelinization, neuronal development, and survival. Binding of ligands to TLRs induces a cascade of intracellular events leading to activation of several transcription factors that regulate the expression of many genes involved in inflammation. During pregnancy, the previous responses promoted by viral infections and other proinflammatory conditions are common and might predispose the offspring to develop psychiatric disorders and neurological diseases. Such disorders could eventually be potentiated by stress and might be part of the etiopathogenesis of CNS dysfunctions including autism spectrum disorders and schizophrenia.

## 1. Introduction

Signaling between nervous and immune systems is in part due to the fact that these two systems share ligands and receptors. The cellular components involved in these interactions within the central nervous system (CNS) are mainly mastocytes, also called mast cells, and glia. In human brain, mastocytes are very scarce and are preferentially located in perivascular territories. By contrast, glial cells comprise about 90% of the total cell content in the CNS and are classified as microglia and macroglia (astrocytes, oligodendrocytes, and ependymal cells) [1]. Representative of the immune system in the CNS are mastocytes and microglia, two cell types derived from hematopoietic cells of the bone marrow that migrate to the brain before closure of the blood brain barrier (BBB) [2, 3]. 

The CNS challenged by different aggressions frequently elicits immune and inflammatory responses [4, 5]. Mastocytes and microglia are efficient sensors of adverse endogenous or exogenous conditions of the CNS [2, 6]. Moreover, stress conditions induce rapid mastocyte degranulation via the hypothalamic peptide corticotropin-releasing hormone (CRH) [7] and exogenous danger molecules like polyinosinic-polycytidylic acid (poly (I:C)), bacterial lipopolysaccharide (LPS), and peptidoglycan (PGN), which are detected by mastocytes and microglia via toll-like receptors (TLRs) [8, 9]. Also, glucocorticoids (GCs) play a relevant role in stress-induced potentiation of neuroinflammatory responses by sensitizing microglia to proinflammatory stimuli [10]. As part of these responses, glial TLRs, connexin hemichannels (Cx HCs), pannexin (Panx) channels might be key players in acute and chronic neurodegenerative diseases characterized by open BBB, demyelinization, and neuronal degeneration [11].

The causes of various chronic diseases that affect the CNS, such as Alzheimer's disease (AD), Parkinson's disease (PD), and multiple sclerosis (MS), are complex and can be related to multiple factors. Notably, the innate host defense has been demonstrated to play an active role in promoting neurodegeneration [12, 13]. However, the possible role of these cellular and molecular elements during brain ontogenesis and the consequences in the adult CNS remain unknown. This review presents possible implications of glial toll-like receptors (TLRs) and Cx HC and Panx channels activation after potentiation by stress in CNS dysfunctions. 

During pregnancy, viral infections are common and emerge to predispose the offspring to develop psychiatric diseases [14, 15]. Viral mimic polyinosinic:polycytidylic acid [poli (I:C)] resembles the structure of double-stranded RNA (dsRNA) generated in host cells during viral replication, and it is recognized by TLR3 that activates the innate immune response [16]. The administration of poly (I:C) is a way to trigger the innate immune response, which mimics the early phase of viral infections [17], and avoids the use of infectious agents, and treatments can be standardized and experiments may be easily compared [18]. All together, they represent an interesting area because perinatal infections, particularly those of viral etiology, are frequent and have been associated with diverse alterations of adult CNS, including schizophrenia and autism [19, 20].

## 2. Toll-Like Receptors (TLRs): Their Expression and Functions in Brain Cells

TLRs are highly conserved germ line-encoded pattern-recognition receptors that initiate innate immune responses via recognition of pathogen-associated molecular patterns (PAMPs) as well by recognition of danger-associated molecular patterns (DAMPS) that correspond to endogenous ligands released after tissue injury or cellular stress, such ATP, histones, heat-shock proteins, mRNA, high-mobility group box-1 protein (HMGB1), surfactant proteins A and D, and mitochondrial proteins [21]. Activation of TLRs triggers a cascade of intracellular events leading to activation of several transcription factors, including NF-*κ*B, activator protein-1 (AP-1), and IFN-regulatory factor-3 (IRF-3) and -7 that regulate the expression of various cytokines and chemokines, responses that are performed in the CNS mainly by mastocytes and microglia. In addition, activation of innate immune responses via TLRs is a prerequisite for the generation of adaptive immune responses [22] that become relevant in autoimmune diseases such as experimental autoimmune encephalomyelitis (EAE). 

The number of molecular members that comprise the TLR family is ten in humans (TLRs 1–10) and twelve in mice (TLRs 1–9; TLRs 11–13) [22]. Some TLRs can be expressed on the cell surface (TLRs 1, 2, 4, 5, 6, and 10) or in intracellular compartments (TLRs 3, 7/8, and 9), but others can be found in both the cell membrane and intracellular compartments (TLR3 and TLR7; endosomes and endoplasmic reticulum) [21]. Each TLR detects distinct PAMPs derived from viruses, bacteria, mycobacteria, fungi, or parasites. For example, TLR3 and TLR7/8 detect ds and single-stranded (ss) RNAs from virus, respectively; TLR4 responds to LPS from Gram-negative bacteria; and TLR9 senses bacterial DNA that contains unmethylated cytosine-guanosine dinucleotides (CpG) [22–25]. 

In the adult brain, mastocytes are mainly found in leptomeninges [2] and thalamus close to the BBB [26, 27], but they are also present early in brain ontogeny [28, 29]. Mastocytes can be activated by antigens that induce crosslinking of IgE bound to mast cells, CD47 recognition, calcium ionophore, ATP, compound 48/80, and also by recognition of DAMPS or PAMPS [26, 27]. If these activators bind to mastocytes for a short period of time (from seconds to a few minutes), they lead to rapid degranulation and release bioamines, proteoglycans, proteases, ATP, TNF-*α* and chemokines stored in preformed granules, whereas activations of longer durations lead to the release of newly formed cytokine (TNF-*α*, IL1*β*, and granulocyte macrophage colony-stimulating factor (GM-CSF)), and chemokine (C–C motif) ligand 3 (CCL3), enzymes (tryptase, chymase, carboxypeptidase), lipid mediators (prostaglandins, leukotrienes, thromboxanes, and platelet-activating factor), and nitric oxide (NO), mediating the recruitment of effector cells, fluid extravasation, and tissue inflammation [30, 31]. 

Murine mastocytes express the mRNA of TLRs 1–4 and 6–9 but not TLR5 [32–36]. Moreover, human mastocytes express the mRNA of TLRs 1–10 with the exception of TLR8 [9, 37–39]. In mastocytes, TLR ligands, such as poly (I:C), LPS, R-848, and CpG oligodeoxynucleotide, promote IL-6 and TNF-*α* secretion as well as regulated upon activation, normal T cell expressed and secreted (RANTES) and macrophage inflammatory protein (MIP) without significant degranulation [35, 38, 40, 41]. More specifically, in rodent mastocytes, binding of LPS to TLR4 induces the release of *de novo* expressed (without degranulation) and secreted TNF-*α*, IL-5, IL-10, and IL-13 but not GM-CSF, IL-1, or leukotriene C4 (LTC4), while binding of PGN to TLR2 induces degranulation that includes histamine release [9, 34, 37].

In three different mouse models, where TLR3, TLR4, and TLR7 were specifically deleted in mastocytes, the recruitment of effector CD8^+^ T cells, neutrophils, and dendritic cells, respectively, was totally avoided after agonist stimulation [33, 42, 43]. This implies that mastocytes recognize, respond, and coordinate immune responses, features that are suppressed by TRLs 3, 4, and 7.

Not only ligands, but also immunological host environments are decisive for mastocyte activity. In human mastocytes, prolonged lymphotoxin-alpha (LTA) and PGN exposure downregulate Fc*ε*RI, decreasing degranulation products after an antigen crosslinking reaction [39]. Poly (I:C) treatment also decreases degranulation in an *in vitro* allergic model, affecting mastocyte adhesion to fibronectin and vitronectin through conformational inactivation of CD29, the receptor of fibronectin [44]. Moreover, LPS and PGN induce mastocytes migration *in vitro* after brief treatment with IL-6 and CCL5/RANTES, respectively [45]. 

The activation and migration of mastocytes occur in several neurologic disorders including MS [46, 47], PD [48], amyotrophic lateral sclerosis (ALS) [49, 50], AD [51], traumatic injury [52], ischemic and hemorrhagic stroke [53, 54], and viral infections [55]. Mastocytes activation and migration are critical for the increased BBB permeability and progression of neuroinflammation. Mastocytes also degranulate upon recognition of myelin basic protein and purinergic P2 receptors [56]. Additionally, proteases released during mastocyte degranulation can also degrade myelin components [57], contributing to myelin damage in the CNS and peripheral nervous system.

Microglia can rapidly respond to pathogens through their TLRs but do not sense apoptotic cells through the same mechanism [58, 59]. They express mRNAs encoding for TLRs 1 to 9. Moreover, levels of TLRs expressed by microglia vary depending on the stages of development or pathological conditions [8]. TLR activation induces a cascade of intracellular events leading to the activation of several transcription factors, including NF-*κ*B, AP-1, IRF-3, and IRF-7 that regulate the expression of many molecular elements of inflammatory responses [60].

In human microglia, activation of TLR3 by agonists such as poly (I:C) induces a strong proinflammatory response that allows microglia to mediate the development of T-helper 1 (Th1) cells [61]. Moreover, infection with the West Nile virus (a retrovirus that produces dsRNA) in mice lacking TLR3 shows reduced microglial activation and more resistance to lethal infection with reduced viral load and inflammatory responses in the brain compared to wild-type mice [62].

Mastocytes release several cytokines in response to TLR2 activation including TNF-*α*, IL-4, IL-5, IL-6, and IL-13. Meanwhile, the activation of TLR4 causes release of TNF-*α*, IL-6, IL-13, IL-5, IL-10, and eotaxin [34, 63–65]. Also, numerous chemokines including CCL5/RANTES, can also induce a proinflammatory profile in microglia [37, 38, 59, 66]. IL-33 derived from microglia modulates the activation of P2 receptors on mastocytes inducing secretion of IL-6, IL-13, and monocyte chemoattractant protein-1 (MIP-1), which in turn can modulate the microglia activity [67]. Tryptase is the main protease secreted by human mastocytes. It is elevated in the CSF of patients with MS [68]. It induces microglia to secrete TNF-*α*, IL-6 [69], and ROS and activate in microglia proteinase-activated receptor-2 (PAR-2), a G protein-coupled receptors widely expressed in neurons, astrocytes, and microglia that are implicated in the pathogenesis of ischemia and neurodegeneration [70], because it induces widespread inflammation [71–73]. The activation of microglial PAR-2 also upregulates P2X_4_ receptors and promotes release of brain-derived neurotrophic factor, TNF-*α*, and IL-6 that upregulate the expression mastocyte of PAR-2, which results in activation and release of TNF-*α* [67].

It is interesting to note that mastocytes but not microglia have been described to be the first responder in CNS injuries, such as perinatal hypoxia-ischemia. Many cells produce TNF-*α* in response to several stimuli, but mastocytes store TNF-*α* in granules, and thus they can release it before other cells including microglia and endothelial cells. Additionally, the recruitment and activation of mastocytes occur previous to responses elicited by neurons, glia, and endothelial cells. Therefore, mastocytes initiate acute inflammations in response to a stimulus, and when inhibited, the brain damage decreases, as observed when the early mastocyte response is inhibited with cromolyn (a mastocyte stabilizer), and then significant neuroprotection is observed [74].

 A strong link between LPS, the TLR4 agonist, and brain injury both in fetal and newborn animals has been demonstrated [75]. LPS injected into developing mouse and rat brains has been shown to induce injury in white matter [76]. Moreover, systemic LPS administration to preterm fetal sheep induces cerebellar white matter injury [77], and *in vitro* assays demonstrate that TLR4 gene deletion prevents LPS-induced oligodendrocyte death [78].

In astrocytes, TLRs mediate the first step of innate immune cell activation. The expression of TLRs is limited in astrocytes, probably because of the neuroectodermal origin of astroglia [79]. These cells express TLR2, which increases in response to proinflammatory stimuli [22, 80]. They also express TLR3 that responds to poly (I:C), hence producing among other cytokines IL-6 that contributes to inflammation in humans and mice [80–82]. The gene profile of astrocytes activated via TLR3 shows neuroprotective mediators and cell growth factors, that is, differentiation and migration molecules comprising a neuroprotective response rather than a proinflammatory phenotype [83, 84].

TLR4 has been shown to participate in stroke-caused brain damage [85–87] and in AD [88, 89]. Likewise, TLR4 could play a pivotal role in demyelinating diseases, such as MS [90]. TLR activation in astrocytes also induces the release of several cytokines and chemokines [91]. Both TLR agonists and cytokines induce the expression of chemokines CCL2, CCL3, CCL5, intercellular cell adhesion molecule-1 (ICAM-1), and vascular cell adhesion molecule-1 (VCAM-1). Moreover, LPS and poly (I:C) induce the production of IL-6, TNF-*α*, IFN-*α*4, IFN-*β*, and iNOS [80]. In addition, poly (I:C) activation induces CXCL-10 production [92]. LPS and dsRNA in parallel induce astrocyte activation, which leads to IL-1*α*, IL-1*β*, IL-6, TNF-*α*, GM-CSF, LT*β*, and TGF-*β*3 secretion, although macrophage migration inhibitory factor (MIF) secretion is inhibited. However, no effect has been found on anti-inflammatory cytokines such as IL-2, IL-3, IL-4, IL-5, IL-10, TGF-*β*1, TGF-*β*2, and TNF-*β* [11, 93].

Recently, in addition to TLR2, TLR3, and TLR4, TLR1, TLR5, TLR6, and TLR7/8 have been found in astrocytes, but their functional roles remain unknown [22, 84]. Therefore, the understanding of the detailed mechanisms of TLR signaling in astrocyte activation in CNS inflammatory conditions still needs further investigation. 

The expression and function of TLRs in oligodendrocytes, unlike other glial cells, have been poorly studied. Only TLR2, -3, and -4 have been evaluated [94], being these receptors related to the regulation of inflammatory processes, gliosis, and remyelination after injury [95, 96]. Knockout mice for TLR2 and TLR4 exposed to spinal cord injuries show a lower remyelination capacity, and thus it is believed that these receptors would have a key role in the formation of myelin [84].

Astrocyte dysfunction triggers primary microglial activation, which induces demyelination [78, 97]. Furthermore, injection of LPS in the bone marrow induces a rapid oligodendrocyte loss, followed by an increase in oligodendrocyte number [98]. After acute demyelination induced by LPS, a more widespread distribution of oligodendrocyte precursor cells is triggered by the activation of microglia/macrophages, which is an event that accelerates remyelination [99, 100].

Rats treated with zymosan, a TLR2 agonist, show oligodendrocyte and axonal loss without regeneration [98]. In addition, rats treated with LPS, that is, a TLR4 agonist, show oligodendrocyte death and demyelination [76, 101]. Also, LPS-induced spinal cord damage shows significant demyelinization associated with an important reduction in the amount of oligodendrocytes [102]. Other researchers have shown that TNF-*α* and TNFR1 play a relevant role in oligodendrocyte death induced by TLR activation [103–105]. However, Bsibsi et al. [100] showed that zymosan and LPS reduce survival, differentiation, and myelin-like membrane formation, while poly (I:C) triggers apoptosis in rat oligodendrocyte cultures. These findings suggest that TLRs play a pivotal role in oligodendrocyte differentiation and myelination, both in physiological and pathological conditions. Compared to other cell types, TLRs play direct roles in regulating various aspects of oligodendrocyte's behavior. However, the apparent contradiction between the effects of LPS and zymosan on oligodendrocytes in different models has not been clarified. Future research could help to determine the functionality of TLR receptors in oligodendrocytes under physiological and pathological conditions.

With regard to the neuroendocrine modulation of the activity of TLRs, this can take local, regional, and systemic routes [106]. Local components include neuropeptides such as substance P, CRH, calcitonin gene-related peptide (CGRP), and endogenous opioids [106] released by peripheral nervous system. Among the regional components, the sympathetic and parasympathetic innervations release neurotransmitters (adrenaline and acetyl choline), and neuropeptides (neuropeptide Y or vasoactive intestinal peptide (VIP)) play a relevant role. Also at a regional level, a neuronal component regulates immunity through the innervation of immune organs and release of noradrenaline, and also a hormonal component regulates immunity systemically by means of adrenaline released from the medulla of the adrenal glands [106], whereas the systemic factors include the neuroendocrine system through the hypothalamic-pituitary-adrenal (HPA) axis and the anti-inflammatory effects of GCs. Furthermore, neuropeptides including cholecystokinin (CCK), somatostatin, melanocyte-stimulating hormone (MSH), VIP, and gastrin also reduce the inflammatory response [107]. 

Additionally, IL-1*β* participates in several aspects of the immune response to infections such as regulation of inflammation and modulation of adaptive immune responses against viral infections [108, 109]. The inflammasome is a multiprotein complex that activates a platform for caspase-1 and caspase-1-dependent proteolytic maturation and secretion of interleukin-1*β* (IL-1*β*). Several inflammasomes have been described being the NLRP3 inflammasome the most extensively studied [110]. It requires two signals. The signal 1 corresponds to TLR ligands or TNF-*α*, and the signal 2 includes ATP, amyloid-*β* (A*β*), K^+^ efflux, pore-forming toxins, and silicic and uric acid crystals [111–113]. After TLR2 and TLR4 activation, secretion and maturation of cytokines IL-1*β* and IL-18 depend on caspase-1 cleavage of their premature forms. In both cases, inflammasome complex proteins mediate caspase-1 activation in the presence of high concentrations of extracellular ATP through activation of P2X_7_ receptors [114, 115]. Activation of P2X_7_ receptor leads to a large membrane pore formation identified as Panx1 channels [116, 117], which recently has been found critical for caspase-1 activation [116, 118]. Not only in immune cells but also in neurons and astrocytes, Panx1 recruitment mediates caspase-1 activation [119], suggesting that during infections, overall TLRs and Panx1 channels could enhance inflammatory responses.

## 3. Cx HCs and Panx1 Channels in Glial Cell and Mastocytes

**Figure 1:**
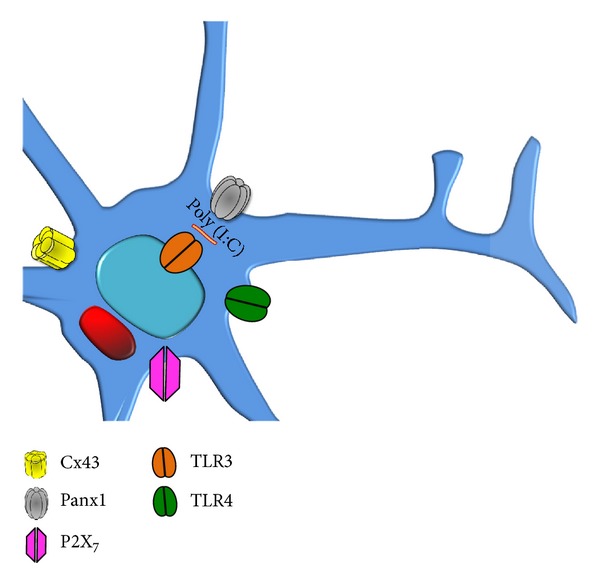
Expression of toll-like receptors in microglia and their relationship with pannexin channels and P2X_7_ receptors. Microglia express TLRs 1 to 9, and in this figure only, TLRs 3 and 4 are shown responding to pathogen-associated molecular patterns of virus (TLR3) and Gram negative bacteria (TLR4). TLR3 is expressed in endosomal membranes (and also in cell surface) and recognizes nucleic acids of virus (dsRNA) and poly (I:C). We propose that microglia under activation with TLR ligands increase the expression of Panx1 channels and connexin HCs and the activity of P2X_7_ receptors.

One HC corresponds to one-half of a gap junction channel and is located at unapposed cell surfaces serving as communication pathway between the intra- and extracellular compartments. Two types of HCs are formed in most cells, and they are generally coexpressed [120]. One of them is formed by connexins (Cxs, 21 in humans) and the other by Panxs 1–3. HCs provide a membrane pathway for releasing signaling molecules (e.g., ATP, glutamate, PGE_2_, and NAD^+^) and thus are recognized as paracrine/autocrine communication pathways under normal and pathological conditions [121, 122]. Inflammation is a key condition in neurodegeneration that occurs in postischemic brain, diabetes, MS, PD, AD, and possibly in various other neurodegenerative diseases [123, 124]. In neuroinflammatory conditions, the successive activation of different glial cells via HCs has been partially demonstrated [125, 126], and mastocytes are likely to be involved in early steps of different pathological conditions (Figure 1). 

As mentioned previously, the degranulation response of mastocytes is an early and rapid response and might require precise coordination where HCs could be essential. Mastocytes express Cxs 32 and 43 [127], but to our knowledge, it remains unknown whether they form functional HCs. In addition, no clear evidence of Panx1 expression in mastocytes has been published, but activation of P2X_7_ receptors leads to the formation of membrane pores permeable to molecules up to about 900 kDa with single currents, similar to what has been described for Panx1 channels, along with histamine release [117, 128]. Since the degranulation process depends on influx of extracellular Ca^2+^ [129], it is possible that Panx1 channels participate in ATP release, and then ATP activates P2X_7_ receptors, which are Ca^2+^ permeable, allowing the influx of Ca^2+^ required for the mastocyte degranulation response. Then, glial cells become involved and microglial cells respond before astrocytes (within several minutes to few hours).

In the normal CNS, microglial cells are in a resting state and are sparsely distributed. They express the macrophage marker CD11b, low levels of CD45, and practically undetectable levels of major histocompatibility complex (MHC) class II molecules, CD40, and CD86. *In vitro*, the microglia activation process is characterized by an upregulation of CD45, MHC class II, and the costimulatory molecules CD40 and CD86 [130, 131]. The expression of MHC II antigens is a characteristic feature of antigen-presenting cells, and their coexpression with costimulatory molecules is a hallmark of microglial cells' ability to interact with other cells, such as T cells. 

Activated, microglia proliferate and migrate to the injury site where they form cell aggregates and secrete pro- and anti-inflammatory cytokines and chemokines, NO, and growth factors [132]. The activation of microglia can be acute or chronic, and this would depend not only on the duration of an external stimulus but also on the quality of the stimulus (stress, infection, inflammation, and signals from damaged neurons) [133]. In fact, they show differences when activation is induced by stress or inflammation. For instance, acute stress induces morphological activation of microglia and increased c-Fos expression in the periaqueductal gray matter but not in the surrounding midbrain. If activation is chronic, it can lead to microglial overactivation followed by microglial degeneration [134]. Therefore, activated microglia secrete TNF-*α* and IL-1*β*, which in astrocytes induce opening of Cx43 HCs leading to the release of ATP and glutamate by astrocytes, which can kill neurons through the activation of Panx1 channels, P2X_7_ receptors, and NMDA receptors in neurons [135].

Another way of cell-cell interaction used by activated microglia can be found in Cx- and Panx-based channels. Microglia express low to undetectable levels of Cx32, Cx36, Cx43, and Cx45 [136–139]. They also express Panx1, and treatment with A*β*_25–35_ has been shown to increase its surface levels [126]. Similarly, the expression of Cx43 is upregulated in cultured rat/mouse microglia treated with LPS or TNF-*α* plus IFN-*γ* [136], calcium ionophore plus phorbol 12-myristate 13-acetate [140], or PGN derived from *Staphylococcus aureus* [139]. However, the possible functional role of Cx-based HCs expressed by activated microglia remains to be elucidated.

Under normal conditions, astrocytes are highly coupled with each other, forming intercellular networks [141], through which Ca^2+^ waves propagate [142]. Extracellular ATP acts as a paracrine messenger in these waves, since it activates purinergic receptors (P2X and P2Y) in astrocytes of surrounding cells, thus resulting in an increase of [Ca^2+^]_*i*_ [143]. The mechanisms for ATP release from astrocytes may include vesicle-mediated exocytosis [144] and diffusion through Cx43 HCs [125, 145, 146] and/or channels formed by Panx1 [147]. Astrocytes also release several transmitters called “gliotransmitters,” including glutamate [148], GABA [149], ATP [150], and adenosine [151]. Increases in [Ca^2+^]_*i*_ can induce the release of gliotransmitters that promote increases in [Ca^2+^]_*i*_ in neighboring neurons, for example, through ATP- and glutamate receptor-dependent pathways [148]. The increased [Ca^2+^]_*i*_ occurs in local astroglia as well as in astrocytes located more distantly. Gliotransmitters might affect diverse neuronal functions including arborization and neuronal plasticity [142] as well as more complex functions such as fear memory [152]. Thus, astrocytic Cx HCs and Panx1 channels might be molecular targets to prevent undesired effects induced by stress.

Most astrocytes also express Cx30 and Cx43 [153], and at least Cx43 forms HCs that are activated by proinflammatory cytokines, hypoxia-reoxygenation, and high glucose [135]. For instance, LPS does not induce cell permeabilization to fluorescent dyes in primary cultures highly enriched with astrocytes of newborn brains, but astrocytes cocultured with microglia respond to LPS with a large increase in Cx43 HC activity [154]. Moreover, the effect of LPS is mimicked by exogenous applied TNF-*α* and IL-*β*, indicating that astrocytes do not respond to LPS in the absence of microglia. Moreover, astrocytes previously exposed for 24 h to medium conditioned by A*β*-treated microglia (CM-A*β*) are permeabilized via Cx43 HCs [126]. As part of the mechanism, TNF-*α* and IL-1*β* have been shown to mimic the effect of CM-A*β*, and neutralizing TNF-*α* with soluble receptors and IL-1*β* antagonists abrogated this effect [125]. Recent *in vivo* studies have demonstrated that Cx43 HCs are critical mediators of postischemic white and gray matter dysfunction and injury [155]. Moreover, upregulation of astroglial Panx1 channels and Cx43 HCs has been found using an experimental model of brain abscess [156], suggesting that both channel types could play an orchestrated function in some inflammatory responses. Cx43 HCs of reactive astrocytes favor the release of excitotoxic compounds, ATP, and glutamate, which activate neuronal P2X_7_ receptors, NMDA receptors, and Panx1 channels, hence promoting neurodegeneration [125]. Activation of neuronal Panx1 channels by ATP and glutamate released through Cx43 HCs from astrocytes exposed to CM-A*β* was shown to induce neuronal death [126]. Therefore, it has been proposed that blockade of astroglia and/or neuronal Cx HCs and Panx1 channels of the inflamed nervous system may represent a strategy to reduce neuronal loss in various pathological states [157–159]. Additionally, the effect of the maternal environment on the developing CNS in the offspring has been analyzed in fetal nonhuman primates. To this end, mothers were subject to a high-fat diet (HFD), and the CNS of the fetuses showed increased levels of IL-1*β* and IL-1 type 1 receptor, as well as a rise in microglia activation markers, suggesting the activation of the local inflammatory response [160]. Under the previous conditions, it is possible that microglia and astrocytes also present upregulation of HC activity, but this needs experimental demonstration. 

Oligodendrocytes might respond within the same time frame as astrocytes, since they can communicate via gap junctions as previously described herein. These cells are responsible for producing and maintaining myelin from the earliest stages of embryonic development to adulthood [161]. Like other cells of the CNS, oligodendrocytes have low renewal capacity [162]. However, oligodendrocyte precursor cells induce remyelination, following the loss of myelin as a consequence of an injury [163]. Many of their functions are accomplished by the expression of a variety of interactions between Cx- and pannexin-based channels. Oligodendrocytes form gap junction channels with cell bodies of adjacent oligodendrocytes and between layers of myelin, called reflective gap junctions [164]; oligodendrocytes form gap junctions with astrocytes as well [165]. Collectively, this gap junction communicated network helps to absorb and remove extracellular K^+^ and glutamate released during neuronal activity, thus generating a spatial buffer where ions and molecules are diluted among cell communicated via gap junction channels [165–167].

The study of demyelinating diseases, consisting of loss or destruction of myelin, has revealed Panx1 channels, Cx HCs, and gap junction channels as key factors in oligodendrocyte survival, as well as neuroprotection and myelin maintenance [168]. Oligodendrocytes express three different connexins: Cx29, Cx32, and Cx47 [169]. Cx32, but not Cx29 or Cx47, is known to form functional HCs. Moreover, by means of the qPCR technique, the mRNA of Panxs 1 and 2 was detected in primary cultures of oligodendrocytes obtained from optic nerves of 12-day-old rats. Both were located in somas as well as in the layers of the myelin sheath [170]. Extracellular ATP mediates the ischemic damage to oligodendrocytes and is partially explained by the activation of Panx1 channels [170]. 

Both genetic and/or inflammatory diseases triggered by viral or toxic sources may affect myelin formation (hypomyelinating diseases) or its maintenance (demyelinating diseases) as it has been found in human diseases associated with HCs formed by mutated Cxs [161]. The first event in pathological manifestations of demyelinating disease of the CNS is the disruption of the BBB that leads to access of demyelinating antibodies [161, 171–174]. Also, activated T cells entering the CNS mediate the release of inflammatory cells, which together with activated microglia release proinflammatory cytokines that promote oligodendrocyte death *in vitro* [175–178]. TNF-*α* binding to its receptor can induce oligodendrocyte apoptosis directly [179]. Indirectly, TNF-*α* and IFN-*γ* can activate microglia and/or macrophage that destroy oligodendrocytes by oxidative stress [180, 181].

Myelin repair occurs after acute inflammatory lesions, such as MS. This repair is called remyelination, and its process, mediated by oligodendrocyte progenitor cells, is associated with functional recovery [163]. It has been shown that chemokine- (CXCL-) 2 and proinflammatory cytokines, such as IL-1*β* and IL-6, promote oligodendrocyte progenitor cell proliferation, differentiation, and remyelination [163]. Under inflammatory conditions, oligodendrocytes show upregulation of MHC I molecules, which are constitutively expressed, as well as Fas, IFN-*γ*, and TNF-*α* receptors (TNFRI-II), transforming them into targets for CD8^+^ cells [175, 176, 182–185]. Under control conditions there is no expression of MHC II molecules in these cells [186, 187]. However, cultured oligodendrocytes treated with IFN-*γ* in the presence of the synthetic GC (dexamethasone) express MHC II molecules [188], suggesting that under stress they could interact with CD4 T lymphocytes and either activate immune reactions or become the targets of T-cell-mediated cytotoxic attack. 

An excess of extracellular ATP is an activator of both innate and acquired immunities, acting as a DAMP that is chemotactic factor for neutrophils, and a strong regulator of activation, death, and survival of microglial cells [189–191]. Pathway for ATP release is highly variable and includes connexin HCs, Panx1 channels, volume-regulated anion channel (VRAC), purinergic P2X_7_ receptor, and/or vesicular exocytosis [192–195]. Moreover, mastocytes represent an abundant source of ATP stored in granules that are released under activation conditions [196–198] such as specific (e.g., IgE + antigen) and nonspecific stimulation (e.g., stress, mechanic stimulation, and osmotic swelling). With regard to the participation of mastocytes in CNS alterations, ATP can be released by trauma-induced degranulation and thus stimulates adjacent neurites via P2X and P2Y receptors. Additionally, the neuropeptide SP released from nerve terminals upon bradykinin stimulation participates in nerve mastocyte communication [199]. This enables interactions between nerve and mast cells and initiates and represents the development of neuroimmunological synapses. Also, glial cells are involved in neuroimmune cross-communication, and ATP induces glial cells to release IL-1*β*, TNF-*α*, and IL-33. Therefore, ATP released from mastocytes is an important autocrine/paracrine/exocrine factor that mediates cross-communication between different cell types [200]. Moreover, human LAD2 mast cells stimulated with IgE, anti-IgE, or substance P (SP) secrete mitochondrial particles, mitochondrial DNA (mtDNA), and ATP in absence of cell death. Furthermore, mitochondria added to mast cells trigger degranulation and release of histamine, PGD_2_, IL-8, TNF-*α*, and IL-1*β*, and this response is partially inhibited by DNAse and ATP receptor antagonists [201].

## 4. Activation of Glial Cells and Mastocytes during Stress and Infection

Only 30 min of immobilization stress can stimulate the HPA axis and cause degranulation in ~70% of rat dura mastocytes [202]. This response could be triggered by neurotensin (NT) and CRH acting on mastocytes increasing the permeability of the BBB [203–206]. As mentioned previously, activated mastocytes release proinflammatory cytokines and ATP among other bioactive compounds that promote microglia, and astrocyte activation and both reactive glia promote neuronal damage [123, 124]. Related to this, acute or chronic stress through GCs sensitizes microglia to a subsequent proinflammatory challenge [10], suggesting that stress should worsen the outcome of neuroinflammation. To our knowledge, it remains unknown if signal transduction of proinflammatory agents via TLRs and activity of HCs is enhanced by GCs or stress. 

Related to the issue presented previously, various neurodegenerative disorders present activation of microglia in different brain regions [124] and restraint combined with water immersion induces massive microglial activation in the hippocampus, hypothalamus, thalamus, and periaqueductal gray matter [207, 208]. Although the precise mechanism of microglia activation induced by stress remains unknown, it is likely that bioactive molecules released by activated mastocytes (see what is mentioned previously) lead to the activation of microglia and, therefore, induce progression of neurodegenerative changes. In an *ex vivo* approach, rats were first pretreated *in vivo* with RU486 (GC receptor antagonist) and then exposed to an acute stressor (inescapable tail shock; IS), and 24 h later, hippocampal microglia were isolated and stimulated with LPS. Microglia obtained from rats not treated with a GC receptor antagonist showed an increase in gene expression of proinflammatory cytokines (IL-1*β* and IL-6). However, in rats pretreated with RU486, the sensitization of microglial to proinflammatory stimuli did not occur [10]. Astrocytic signaling is potentiated by GCs (i.e., methylprednisolone and dexamethasone) via long-range calcium waves, and an increase is observed in resting cytosolic Ca^2+^ levels, as well as the extent and amplitude of calcium wave propagation (twofold) compared to control conditions [209]. Furthermore, it is known that stress affects microglial function and viability during adulthood and early postnatal life [210]. Experiments both *in vitro* and *in vivo* have shown that stress hormones can affect the function and viability of microglia. However, little is known if stress during pregnancy affects microglia of the offspring. In a recent report, prenatal stress effects on microglia of the offspring were studied. In this model, prenatal stress during embryonic days 10–20 consisted of 20 min of forced swimming. In the offspring, a reduction in the number of immature microglia in the two main brain reservoirs of amoeboid microglia, corpus callosum, and internal capsule was observed. Moreover, accelerated microglial differentiation into ramified forms in the internal capsule and brain regions, such as the entorhinal cortex, parietal lobe neocortex, thalamus, and septum, was seen in the neonates in relation to an increase in plasma corticosterone in the pregnant dam [211].

The stimulation of microglial TLR3 with its ligand leads to the release of IL-6, IL-12, TNF-*α*, and IFN-*γ* among others (Figure 1). In connection to this, the importance of TLR in various CNS diseases (i.e., infection, trauma, stroke, neurodegeneration, and autoimmunity) has been described [212]. This is how viral infections have been implicated in the onset of MS by stimulation of TLR3 [105]. Additionally, in an animal model of schizophrenia, the stimulation of pregnant mothers with poly (I:C) results in reduced neuronal arborization of the offspring, which is correlated with a status of higher activation [213]. Interestingly, Cx HCs participate in neurite outgrowth [214] and release of ATP and glutamate [125], which also affect neuronal arborization [214, 215].

It is interesting to note that sensitivity to drug abuse behavior, as well the neuroinflammatory response to a subsequent proinflammatory challenge (as noted previously), is associated with stress and stress-induced release of GCs. Neuroinflammatory mediators derived from glia have an important role in the development of drug abuse [216]. This is how neuroinflammatory mediators, such as proinflammatory cytokines, are induced by opioids, psychostimulants, and alcohol, all of which modulate many effects including drug reward, dependence, tolerance, and analgesic properties. An interesting aspect is that drugs of abuse may directly activate microglial and astroglial cells via TLRs, which mediate the innate immune response to pathogens [216]. A key aspect is the timing of stress exposure relative to inflammatory challenge, and if a proinflammatory stimulus (e.g., LPS) is added immediately before stress exposure, stress induces an anti-inflammatory effect, which is reflected in the inhibition of the increase in brain IL-1*β* levels [217].

The importance of stress associated with infections is given by the fact that the acute or chronic stress sensitizes the inflammatory responses of the CNS to immunological challenges. Microglia show an increase in expression of MHC II, TLR4, and the F4/80 antigens. Therefore, stress changes the microenvironment of the CNS to a phenotype with inflammatory characteristics. One explanation to this phenomenon is that GCs sensitize microglia to infections [10, 218]. In peripheral blood monocytes from individuals under chronic stress, an increase in the expression of genes with promoter response elements for NF-*κ*B is observed as well as allows expression of genes that have promoter elements for GC receptors [219]. Otherwise, in older stressed or chronically depressed adults, an increase in inflammatory response occurs when they are challenged with antigens, showing depressive characteristics and elevated levels of IL-6 after immunization with influenza vaccines. Further evidence that supports this notion comes from observations in older caregivers of patients with dementia, who also presented an elevation of IL-6 for over four weeks after vaccination with influenza vaccines, whereas this elevation was not observed in non stressed individuals [220].

Furthermore, stress worsens immunity and brain inflammation, which is important in MS and neuropsychiatric disorders [221–226]. Under stress, the neuropeptides CRH and NT are secreted and thus can activate microglia and mast cells, which in turn release molecules with proinflammatory properties. This results in maturation and activation of Th17 autoimmune cells and disruption of the BBB that leads to T cells entry into the CNS enhancing the brain inflammation, which might support the pathogenesis of MS. NT also stimulates secretion of vascular endothelial growth factor (VEGF) and induces expression of CRH receptor-1 in mast cells [20, 206, 227]. Several lines of evidence associate microglia with the pathogenesis of MS because activation of microglia is prominent and precedes T-lymphocyte infiltration and demyelination [228]. Activated microglia release glutamate and NO causing neuronal death and BBB disruption [228, 229]. With regard to the participation of mastocytes in the pathogenesis of MS, patients with this disease show elevated levels of tryptase (that activate microglia) and histamine in cerebrospinal fluid (CSF) [68, 230]. Therefore, several lines of evidence suggest an important role of mastocytes and microglia in neuroinflammatory diseases [67]. Therefore, both cell types represent therapeutic targets to be considered for treatment of MS and other neuroinflammatory diseases.

Among the factors relevant to the development of autism spectrum disorders (ASD), stress during pregnancy and the first 6 months of postnatal life has been associated with increased risk of ASD [231]. Stress induces the secretion of CRH from the hypothalamus and activates the HPA axis [232]. As mentioned previously, CRH also activates mast cells, resulting in the release of several proinflammatory cytokines [233] including IL-6, which in turns may increase the BBB permeability [222, 234, 235].

Recently, a decrease in the mitochondrial function in approximately 60% of patients with autism has been demonstrated [236–238]. The brain of these patients shows lines of evidence of neuroinflammation [239–242], with high levels of mitochondrial DNA [243]. Additionally, elevated levels of NT that could activate mast cells have been detected in children with autism [244]. The involvement of mast cells and brain inflammation is related to mitochondrial fission and translocation to the cell surface during degranulation [245], which leads to release of ATP and mitochondrial DNA [243]. The importance of ATP is that it can maintain inflammation by activating mast cells [225, 246].

## 5. Concluding Remarks

Stress potentiates neuroinflammatory responses by sensitizing microglia to proinflammatory stimuli [10]. This is how prenatal stress modifies the phenotype, distribution, and activation statuses of microglia in the offspring [211]. Different stressors, together with the activation of the inflammatory immune response, enhance the effects of proinflammatory molecules or conditions, showing synergistic effects [247]. Viral infections are the most common causes of infection during prenatal life, and maternal respiratory infection can also increase the risk of the offspring to develop certain mental disorders. The most direct evidence for this comes from a prospective study of pregnant women with medically documented respiratory infections, where the risk for schizophrenia in the offspring is increased 3-fold by infection in the second trimester [248]. Evidence that supports this phenomenon comes from models of cocultures between astroglia and microglia treated with dexamethasone. In these experiments, functional membrane properties of astrocytes in cocultures are differentially regulated, which might reflect steroid effects in adjacent glial components *in vivo*. In cocultures with 30% microglia, dexamethasone-treated cocultures show significant increased gap junctional intercellular communication [249], which could facilitate the propagation of inflammatory signal along astrocytic networks. Therefore, if a stressor is sufficiently sustained, this may reflect neurochemical processes that can make the organism more vulnerable to pathological stimuli producing behavioral and neurochemical responses [250, 251]. This can be reflected in an increased susceptibility to diseases of the nervous system, such as the progression of depressive disorders and anxiety, and can even affect the course of neurological diseases [250, 251]. Furthermore, activated microglia affect the expression of Cx HCs in astrocytes, which in turn increases the astrocytic ATP and glutamate release with deleterious consequences on neurons [125]. Therefore, these lines of evidence represent an aspect to be addressed in a model of stress in pregnant animals, in which one can analyze the effects of stress on microglia of the offspring in terms of activation and its effect on astrocytes, which could promote neuronal damage, with Cx HCs and Panx1 channels being possible therapeutic targets. Additionally, the synergistic effect of stress and stimulation with viral infection (for which RNA viral mimics poly (I:C)) has not been studied in offspring of pregnant females subjected to stress, which is also a novel approach and can be correlated with a possible susceptibility of offspring to diseases of the nervous system. 

An important aspect is that when microglia are strongly activated, they remain in a preactivate state for years, which means that microglia are excessively responsive to even slight stimuli. This fact also has been linked to the activation of microglia by viral infections early in life and that can be later reactivated more rapidly compared to microglia in normal state [252, 253]. Therefore, the possibility of having microglia (using minocycline) and mastocytes activation (with GRH-R antagonists) as therapeutic targets opens the possibility of their modulation as treatment for various neuropsychiatric disorders, viral infections, and other neuroinflammatory pathologies of the CNS.

**Figure 2:**
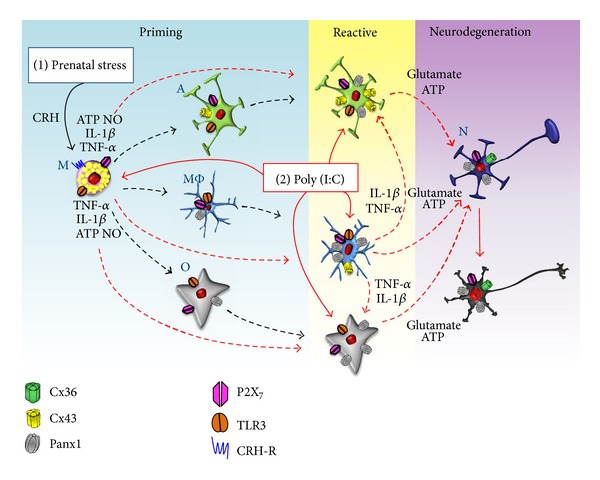
Model of the involvement of mastocytes and microglia in neuroinflammatory responses and potentiation of their responses by stress. Stress increases the levels of CRH and glucocorticoids which are critical players in stress-induced mastocytes (M) degranulation and potentiation of glial inflammatory responses (sensibilization). Furthermore, perinatal infections, particularly those of viral etiology (poly (I:C)), are frequent and have been associated with diverse alterations of CNS. Mastocytes and microglia (MΦ) express toll-like receptor 3 (TLR3). Activated microglia and mastocytes increase the hemichannel activity in reactive astrocytes (A) and oligodendrocytes (O). Both activated microglia and astrocytes release ATP and glutamate that induce neurodegeneration through the activation of P2X_7_ receptors and Panx1 channels in neurons (N) (neurodegeneration) (modified from Orellana et al., 2011) [125].

In summary, parental stress is proposed to induce potentiation of neuroinflammatory responses by first: activating directly mast cells through CRH recognition. Second: mast cells proinflammatory mediators prime microglia, astrocytes and olygodendrocytes, modifying their phenotype, distribution, and activation statuses in the offspring, but mainly promoting HC expression. Third: sensitized microglia exposed to inflammatory stimuli (i.e., TLR3 ligands) (Figure 1) are activated and secrete cytokines (TNF-*α*, IL-1*β*). They also show increased functional expression of Panx1 channels and Cx HCs through which ATP and glutamate are released to the extracellular milieu. Astrocyte and oligodendrocyte become activated and release ATP and glutamate in an HC depending way, and thus they promote neurodegeneration (Figure 2). Therefore, HCs represent a novel target with clinical applications in neuroinflammatory diseases.
